# Distinct Associations of Cognitive Impairments and Reduced Gray Matter Volumes in Remitted Patients with Schizophrenia and Bipolar Disorder

**DOI:** 10.1155/2020/8859388

**Published:** 2020-12-10

**Authors:** Ting Sun, Pengfei Zhao, Xiaowei Jiang, Yifang Zhou, Chao Li, Linna Jia, Yanqing Tang

**Affiliations:** ^1^Brain Function Research Section, The First Affiliated Hospital of China Medical University, Shenyang, Liaoning 110001, China; ^2^Department of Psychiatry, The First Affiliated Hospital of China Medical University, Shenyang, Liaoning 110001, China; ^3^Department of Radiology, The First Affiliated Hospital of China Medical University, Shenyang, Liaoning 110001, China; ^4^Department of Geriatric Medicine, The First Affiliated Hospital of China Medical University, Shenyang, Liaoning 110001, China

## Abstract

**Background:**

Cognitive impairments are documented in schizophrenia (SZ) and bipolar disorder (BD) and may be related to gray matter volumes (GMVs). Thus, this study is aimed at exploring whether the association between cognitive impairments and GMV alterations is similar in patients with SZ and BD and understanding the underlying neurobiological mechanisms.

**Methods:**

A total of 137 adult subjects (46 with SZ, 35 with BD, and 56 age-, sex-, and education-matched healthy controls (HC)) completed the MATRICS Consensus Cognitive Battery (MCCB) and structural magnetic resonance imaging scanning. We performed group comparisons of the cognitive impairments, the GMV alterations, and the association between them.

**Results:**

Compared with HC, the patients with SZ and BD showed shared deficits in 4 cognitive domains (i.e., processing speed, working memory, problem solving, and social cognition) and the composite. SZ and BD had commonly decreased GMVs, mainly in the insula, superior temporal pole, amygdala, anterior cingulate, and frontal cortices (superior, middle, opercular inferior, and orbital frontal gyrus). No correlation between MCCB scores and GMVs was detected in SZ. However, for BD, working memory was relevant to the right hemisphere (i.e., right insula, amygdala, superior temporal pole, and medial and dorsolateral superior frontal gyrus). *Limitations*. The major limitations were that not all patients were the first-episode status and no medication.

**Conclusions:**

The association was mainly limited to the BD group. Thus, the underlying pathophysiology of the cognitive deficits, in terms of GMV alterations, may be diverse between two disorders.

## 1. Introduction

Cognitive impairments are the characteristic of schizophrenia (SZ) [1], covering almost all main domains. Although not as severe as those with SZ [2–4], patients with bipolar disorder (BD) also suffer significantly and share considerable overlaps with SZ in several cognitive domains, especially processing speed, verbal learning, and working memory [5, 6]. Impairments persist even in the absence of affective and/or psychotic symptoms [6–8], thereby seriously affecting sociooccupational ability and causing these clinically stable people (remitted SZ and BD) to remain unable of having normal or relatively normal social life [1, 6, 7, 9].

A series of studies, such as neuroinflammation [10, 11] and neurotrophic factor [12, 13], has been conducted on the impaired cognitive function of SZ and BD, but the underlying neurobiological mechanism is still unclear. Neuroimaging techniques, applied universally in the study of neuropsychiatric disorders, infer alterations of brain structure may have an impact on cognitive function [14, 15]. Voxel-based morphometry is a useful method in investigating the whole-brain structural alterations [16].

Many findings on the altered gray matter volumes (GMVs) of subjects with SZ and BD have been reported. Although the results of these reports have slight differences, similar alterations were observed in patients with SZ and BD. For example, one study reported changed GMVs in multiple frontal-temporal cortices of the patients with SZ across two cultural backgrounds (Germany and Japan) [17]. Meanwhile, other authors used meta-analyses to summarize GMV alterations in BD and also informed the regions located in frontal-temporal cortices [18]. These common brain structural alterations were supported by the findings of other researchers [19, 20]. Other similarly altered GMVs in patients with SZ and BD, such as cingulate and insula, were also documented [19–22].

These GMV alterations reported above are associated with cognitive impairments in subjects with SZ and BD. For instance, small frontal GMVs are associated with low premorbid intelligence quotient in patients with SZ and BD [15, 23]. However, studies on the association are limited thus far, and differences were observed in the findings, which were mainly concentrated on the following aspects: (1) the same impaired domain is associated with different GMV reductions in two disorders, such as social cognition, which is linked to the medial prefrontal cortex in SZ [24], while it is connected to the right middle cingulate gyrus in BD [25]; and (2) the two fields do not correlate, as the results of a cross-sectional study in subjects with SZ, which revealed that metacognition ability is independent of GMV alterations [26], and as the findings in those with BD, which indicated that cognitive deficits and GMVs have no association [27, 28].

These contradictory findings should be further studied to advance the understanding of altered brain structure that is linked to cognitive deficits. Considering the effect of the mood state and/or psychotic symptoms on GMVs [29], we focused on remitted patients with SZ and BD. We supposed that shared cognitive deficits and common GMV alterations in subjects with SZ and BD had similar associations. Thus, this study is aimed at determining similarities between the two patient groups in the severity of cognitive deficits, the extent of GMV alterations, and the correlation between cognitive impairments and GMV changes and subsequently at understanding the underlying neurobiological mechanisms of cognitive impairments in psychiatric disorders.

## 2. Materials and Methods

### 2.1. Participants

The study was conducted in a single site and recruited 137 individuals (age range, 18–50 years old): 46 with SZ, 35 with BD, and 56 healthy controls (HC). After a detailed description of the present study, all participants provided written informed consent as approved by the Medical Science Research Ethics Committee of the First Affiliated Hospital of China Medical University. All participants were recruited from the inpatient and outpatient services at the Shenyang Mental Health Center and the Department of Psychiatry, First Affiliated Hospital of China Medical University, Shenyang, China. HC was recruited from the surrounding community via advertisement. The presence or absence of Axis I psychiatric diagnoses in participants was determined by two trained psychiatrists via the Structured Clinical Interview for Diagnostic and Statistical Manual of Mental Disorders, Fourth Edition (DSM-IV) Axis I Disorders. All patients met the DSM-IV diagnostic criteria for BD or SZ without any other Axis I disorders. HC had no current or lifetime, personal or familial history of DSM-IV Axis I disorders. Exclusion criteria for all participants included the following: (1) substance/alcohol abuse or dependence, (2) any concomitant major medical disorder, (3) any neurological illness, (4) a history of head trauma with loss of consciousness for ≥5 min, (5) any magnetic resonance imaging (MRI) contraindications, and (6) suboptimal imaging data quality.

Symptom severity was assessed by the Hamilton Depression Rating Scale (HAMD-17) [30], Young Mania Rating Scale (YMRS) [31], and Brief Psychiatric Rating Scale (BPRS) [32]. The clinically stable criteria for patients included the following: (1) for SZ: BPRS score < 35; and (2) for BD: YMRS score < 7 and HAMD − 17 score < 7.

### 2.2. Cognitive Assessment

The Measurement and Treatment Research to Improve Cognition in Schizophrenia (MATRICS) Consensus Cognitive Battery (MCCB) is a reliable tool in assessing cognitive function from multidomains, which was introduced to evaluate and promote cognition in SZ and validated subsequently in BD [33–36]. The MCCB contains 10 tasks across 7 cognitive domains, including (a) processing speed (Trail Making Test A, Symbol Coding, and Category Fluency), (b) verbal learning (Hopkins Verbal Learning Test-Revised), (c) working memory (Spatial Span, Letter Number Span), (d) visual learning (Brief Visuospatial Memory Test-Revised), (e) reasoning, problem solving (The Mazes), (f) attention-vigilance (Continuous Performance Test-Identical Pairs), and (g) social cognition (Mayer–Salovey–Caruso Emotional Intelligence Test). A total of 10 subtest scores and a composite score are included in this instrument. All subjects were tested cognition on the same day as the MRI scan.

### 2.3. MRI Acquisition

MRI scans were performed on a GE Signa HD 3.0-T scanner (General Electric, Milwaukee, USA) at the Image Institute of the First Affiliated Hospital of China Medical University, Shenyang, China. T1-weighted, high-resolution, and 3D image data were collected using a 3D fast spoiled gradient-echo sequence (repetition time = 7.2 ms, echo time = 3.2 ms, field of view = 240 × 240 mm^2^, matrix = 240 × 240, flip angle = 13°, slice thickness = 1 mm, number of slices = 176, no gap, voxel size = 1.0 mm^3^). A standard head coil was applied to transmit and receive the nuclear magnetic resonance signal, while earplugs and foam pads were used to reduce noise and head motion. During scanning, subjects were informed to keep their eyes closed but warned not to fall asleep.

### 2.4. Data Processing

As suggested by the forerunners [37], we used the voxel-based morphometry (VBM8) toolbox (http://dbm.neuro.uni-jena.de/vbm8/) to process the structural MRI data, which were incorporated into the Statistical Parametric Mapping (SPM8) software. The VBM8 processing steps included bias correction, tissue segmentation, and spatial normalization (Montreal Neurological Institute space, resampled to 1.5 mm^3^ isotropic voxels) by using Diffeomorphic Anatomical Registration Through Exponentiated Lie algebra (DARTEL) [38], modulation process (nonlinear deformations), and smoothing (Gaussian kernel with 8 mm full width at half-maximum).

### 2.5. Statistical Analysis

Three groups' (SZ, BD, and HC) analyses of the demographic and clinical data were performed in the SPSS 20.0 software (SPSS Inc., Chicago, Illinois) using one-way analysis of variance (ANOVA), independent-samples *t*-test, or the Kruskal-Wallis test for continuous variables, and the Chi-square test for categorical variables. The MCCB scores among three groups were also compared in SPSS, using a one-way analysis of covariance (ANCOVA) with diagnosis as an independent factor, and gender and age as covariates. The GMVs were analyzed in SPM8 and Data Processing Assistant for Resting-State fMRI (DPABI, 2.3, Advanced edition), and ANCOVA was also used with diagnosis as an independent factor, and gender and age as covariates. To determine the significant brain regions statistically, which were identified by the Anatomical Automatic Labeling (AAL) template, we set *p* < 0.001 for each voxel and *p* < 0.05 for multiple comparisons (Gaussian random field (GRF) correction). GMVs were extracted from these significantly different regions and compared between each pair (*p* < 0.05, Bonferroni correction). Then, the partial correlation analysis was employed to analyze the relationship between abnormal MCCB scores and extracted significantly GMV values with gender and age as controlled factors, and the significance level at *p* < 0.05 (false discovery rate (FDR) correction).

## 3. Results

### 3.1. Demographic and Clinical Analyses

Among the SZ, BD, and HC groups, the differences in age, gender, and education were not significant, and all participants were right-handed. For scale scores, significant group effects were found in HAMD-17 (*p* = 0.001) and BPRS (*p* < 0.001), but not YMRS among the three groups. The duration of the illness and the proportion of the medication use were not significantly different between the SZ and BD groups, but the type of drug was different. The SZ group used more antipsychotic drugs (*p* = 0.007), while the BD group utilized more mood stabilizers (*p* < 0.001). The first-episode status was different between the two patient groups (*p* = 0.007). More analyses about the first-episode status and medication are listed in the supplemental file (Tables S1–S6). Details about demographic and clinical data are presented in Table 1.

### 3.2. Cognitive Assessment Results

**Figure 1:**
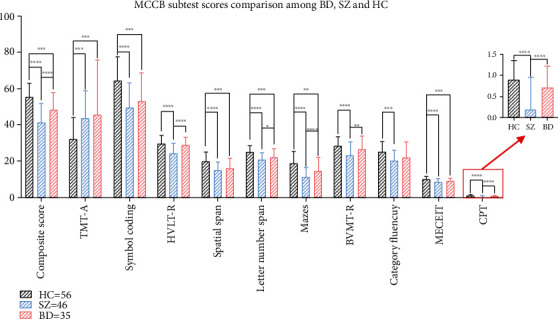
MCCB subtest scores comparison among BD, SZ and HC. TMT-A, Trail Making Test A; HVLT-R, Hopkins Verbal Learning Test-Revised; BVMT-R, Brief Visuospatial Memory Test-Revised; MSCEIT, Mayer–Salovey–Caruso Emotional Intelligence Test; CPT-IP, Continuous Performance Test-Identical Pairs. Note: ∗*p* < 0.05, ∗∗*p* < 0.01, ∗∗∗*p* < 0.005, ∗∗∗∗*p* < 0.001.

ANCOVA showed significant differences in cognitive function among the three groups. First, the composite score of the patients with SZ and BD was lower than that of the HC subjects, but SZ manifested worse than BD. For MCCB subtests, post hoc analyses revealed that compared with HC, the SZ group showed impairments in all 10 cognitive tasks, while the deficits of the BD group were detected in 6 tasks, including Trail Making Test A (TMT-A), Symbol Coding, Spatial Span, Letter Number Span, Mazes, and Mayer–Salovey–Caruso Emotional Intelligence Test (MSCEIT). Finally, the distinction between patients with SZ and BD in the Hopkins Verbal Learning Test (HVLT-R), Letter Number Span, Mazes, Brief Visuospatial Memory Test-Revised (BVMT-R), and Continuous Performance Test-Identical Pairs (CPT-IP) was significant (*p* < 0.05, Bonferroni correction; Figure 1).

### 3.3. Differences in GMV Groups

**Figure 2:**
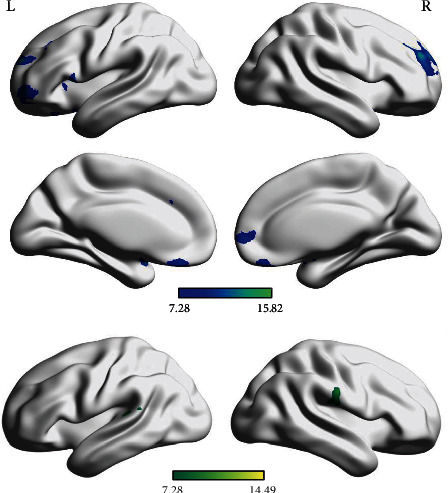
GMV alteration among BD, SZ, and HC. Significant at *p* < 0.05 with voxel *p* < 0.001 (GRF correction). Blue colour indicates relatively lower GMVs values in both BD and SZ. Green colour indicates relatively lower GMVs values in SZ.

Significant group effects were detected in 10 clusters across 17 brain regions. Compared with HC, SZ and BD groups had decreased GMVs in the bilateral insula, bilateral temporal pole-superior temporal gyrus (TPOsup), limbic system (right amygdala and left anterior cingulate and paracingulate gyri (ACG)), as well as several regions of the frontal cortices (bilateral dorsolateral superior frontal gyrus (SFGdor), right medial superior frontal gyrus (SFGmed), left middle frontal gyrus (MFG), left opercular inferior frontal gyrus (IFGoperc), left orbital middle frontal gyrus (ORBmid), left orbital superior frontal gyrus (ORBsup), and bilateral rectus (REC)). Besides, there were 2 regions (i.e., left superior temporal gyrus and right supramarginal gyrus) reduced only in the SZ group (*p* < 0.05, Bonferroni correction; Table 2 and Figure 2).

### 3.4. Correlation between GMVs and MCCB

**Figure 3:**
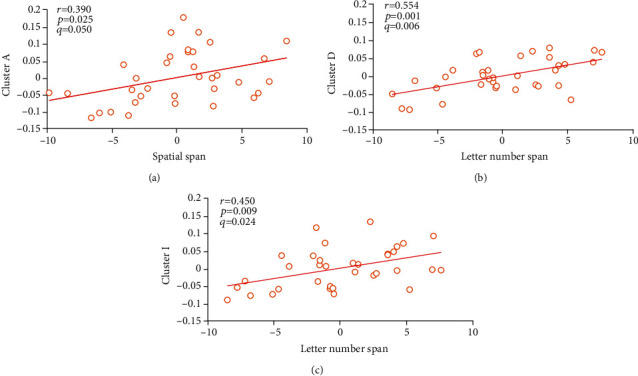
Correlations between MCCB scores and GMVs in BD. Significant at *q* < 0.05, FDR correction.

No correlation was observed between MCCB scores and GMVs in subjects with SZ anywhere across 17 altered brain regions. Nevertheless, a series of links were detected in the BD group, concentrating on the cognitive subtest across Spatial Span and Letter Number Span, which comprised working memory. Specifically, the scores of Spatial Span were relevant to the right SFGdor, and the scores of Letter Number Span were affected by the right insula, amygdala, TPOsup, and SFGmed. (*q* < 0.05, FDR correction; Figure 3).

## 4. Discussions

This study focused on probands with remitted SZ and BD, from cognitive impairments, GMV alterations, and the correlation between them, in which substantial similarities and differences were observed.

Considering the composite cognition, all patients with SZ and BD had deficits, and the performance of SZ was poor, which was consistent with the findings of prior studies on cognitive deficits [4, 39], wherein SZ has a worse composite score than BD. In terms of every cognitive subtest of MCCB, the SZ group showed impairments in overall 7 cognitive domains, whereas the BD group presented cognitive impairments only in 4 domains, that is, processing speed, working memory, problem solving, and social cognition, just like the findings of several cross-sectional studies [6, 40, 41]. However, some studies have found other impaired cognitive domains in patients with BD, such as deficits in visual and verbal learning found by Van Rheenen and Rossell [34]. Another study that selected BD patients during the onset found no problem solving or social cognitive abnormalities [35]. These conflicting results may be due to the emotional states of patients which were different from our study. Additionally, the BD group performed better than the SZ group in verbal learning, working memory, problem solving, visual learning, and attention, which was similar to the result of another study that concentrated on the verbal episodic memory of SZ and BD [42]. According to the findings above, the cognitive impairments of patients with SZ were more severe than those of patients with BD, broad consent with previous studies [36, 43].

Regardless of probands with SZ or BD, the alterations of GMVs in every brain region which was discovered differences from HC were reduced. This result was supported by other authors. For example, several authors documented the entire cortex volume reductions in patients with SZ who had cognitive impairments [44], and others reported that cognitively impaired patients with SZ and BD exhibited small total brain volumes [45]. Relative to HC, the common brain structural changes in both patient groups were mainly concentrated on 4 areas, including insular, temporal cortex (bilateral TPOsup), limbic system (right amygdala and left ACG), and frontal cortices (bilateral SFGdor, right SFGmed, left MFG, left IFGoperc, left ORBmid, left ORBsup, and bilateral REC), which was consistent with the results of a review that summarized the findings of GMV comparisons between SZ and BD and pointed overlapping reductions in the insula and ACG [46], and following the results of a matched control study that indicated small GMVs within frontal and temporal regions in both SZ and BD [47]. This result also agreed with the findings of other studies [21, 25, 29]. Besides, as early studies detected, our study also reported that GMV damages in patients with SZ covered more areas than those with BD, that is, primarily left STG and right SMG [46, 48].

The results of the correlation analysis showed that only the BD group had associations between cognitive impairments and GMV alterations. The decreased working memory of BD was related to reduced GMVs of the right hemisphere, containing the right insula, amygdala, TPOsup, SFGmed, and SFGdor. However, the study on the association between cognitive deficits and GMV damages in patients with BD was limited. There was a study on the gray matter density of pediatric patients with BD, in which the reductions of the left orbitofrontal cortex are associated with working memory [49]. The differences from us may be due to the age range of the participants, because the gray matter of the child is still in the developmental stage. As for other cognitive impaired domains of BD, they were independent of GMV alterations, including processing speed and social cognition, against previous surveys.

However, the probands with remitted SZ, who had more cognitive impairments and GMV alterations, were undetected any association between MCCB subtest scores and extracted values from changed brain regions. This result was different from those of prior studies. For example, one study in a Chinese Han population with SZ reported impaired working memory was correlated with GMV reductions and fractional anisotropy decrease in prefrontal and superior temporal area [50], but they applied a different method—multimodal fusion, to measure brain abnormity. Another study informed the link between hippocampal subregion volumes and cognitive performance in visual, verbal, and working memory [51], whereas we found no alterations around hippocampal. Because we aimed to identify neuroimaging substrates of cognitive impairments in psychiatric disorders, other nonaltered regions were not performed a correlation analysis, which may also be associated with cognition but not related to the diseases. Meanwhile, the findings of a Japanese research suggested that the anterior cingulate and medial frontal cortices volumes affect working memory in SZ [52]. Divergence may be considered since the patients included in the study were disease-onset, whereas the mood state and/or psychotic symptoms have an effect on GMVs [29]. Another important reason for the differences from others was that we used partial correlation analysis with gender and age as controlled factors, so that the results were net and little.

Whether the neurobiological mechanism behind SZ and BD cognitive impairment is consistent has been controversial. In recent years, some researchers propose a continuum between SZ and BD [53], so that the mechanism of the two diseases should be the same. However, our results supported the traditional view that the two diseases are independent of each other. Some studies have explored the mechanism by functional magnetic resonance imaging or white matter integrity [52, 54], and the results are also divergent. There cannot be a conclusion on this matter whether cognitive impairment is caused by a single lesion or multiple lesions, yet in terms of our results, the patterns of SZ and BD were different.

Overall, this is the first study that focused on probands with remitted SZ and BD from the neurobiological mechanism behind cognitive impairment, using MCCB to assess cognitive function and GMVs to measure brain structural alterations. We eliminated the effects of the mood state and/or psychotic symptoms by strictly limiting the state of the disease. Our results added meaningful evidence for the study of cognitive impairment mechanisms in psychiatric illnesses.

## 5. Limitations

The major limitations of the study were the effects of the first-episode status and medication on cognition and GMV alterations. After analyzing these factors in the two patient groups, we made some findings. The first-episode status and the use of antipsychotic affected the working memory of both patient groups. The effect of mood stabilizer was only on the GMV alterations in the SZ group. Details were listed in the supplemental file (Tables S1–S6). Hence, additional large-scale surveys with strict limitations are needed.

## 6. Conclusions

SZ and BD groups had shared cognitive impairments and GMV alterations, but the SZ group was more severe than the BD group in both fields. The association between the two fields was mainly limited to the BD group. Consequently, the underlying pathophysiology of cognitive deficits, at least brain structure, may be diverse between two disorders.

## Figures and Tables

**Table 1:** Demographic and clinical characteristics.

Characteristic	Group; mean ± SD or no. (%)			*F*/*χ*^2^/*t*/*H*	*p* value	Post hoc analysis
	HC (*n* = 56)	SZ (*n* = 46)	BD (*n* = 35)			
Age, year^a^	29.54 ± 9.41	29.70 ± 8.90	32.20 ± 10.50	0.966	0.383	—
Male sex^b^	21 (37.5%)	13 (28.3%)	10 (28.6%)	1.260	0.533	—
Education, year^a^	14.48 ± 3.30	12.96 ± 3.03	13.83 ± 3.37	2.821	0.063	—
Handedness, right^b^	56 (100%)	46 (100%)	35 (100%)	—	—	—
First episode, yes^b^	—	27 (58.7%)	10 (28.6%)	7.269	0.007∗	SZ>BD
Duration (month)^c^	—	42.15 ± 55.36 (*n* = 40)	56.81 ± 57.43 (*n* = 32)	1.071	0.304	—
Medication, yes^b^	—	42 (91.3%)	29 (82.9%)	1.311	0.252	—
Antipsychotic^b^	—	38 (86.4%)	17 (58.6%)	7.242	0.007∗	SZ>BD
Mood stabilizer^b^	—	7 (15.9%)	17 (58.6%)	14.450	<0.001∗	BD>SZ
Antidepressant^b^	—	11 (25.0%)	6 (20.7%)	0.182	0.670	—
HAMD-17, total score^d^	1.11 ± 1.53	3.27 ± 3.98	2.37 ± 2.29	13.537	0.001∗	SZ>HC (*p* = 0.002∗) BD>HC (*p* = 0.024∗) SZ vs. BD (*p* = 1.000)
YMRS, total score^d^	0.18 ± 0.61	0.67 ± 1.62	0.63 ± 1.57	3.274	0.195	—
BPRS, total score^d^	18.54 ± 1.04	21.93 ± 4.35	20.94 ± 5.22	24.500	<0.001∗	SZ>HC (*p* < 0.001∗) BD>HC (*p* = 0.003∗) SZ vs. BD (*p* = 0.186)

BD, bipolar disorder; BPRS, Brief Psychiatric Rating Scale; *F*, one-way ANOVA; *H*, Kruskal-Wallis test; HAMD-17, Hamilton Depression Scale; HAMA, Hamilton Anxiety Scale; HC, healthy control; SZ, schizophrenia; SD, standard deviation; *t*, independent-samples *t*-test; YMRS, Young Mania Rating Scale; *χ*^2^, Chi-square test; ^a^One-way ANOVA; ^b^Chi-square test; ^c^independent-samples *t*-test; ^d^Kruskal-Wallis test. ∗Significant at *p* < 0.05; post hoc analysis is the Bonferroni correction.

**Table 2:** Clusters showing significant differences across BD, SZ, and HC groups with one-way ANCOVA.

Cluster	Brain regions	Voxels	Peak MNI coordinate			*F*	*p*	Post hoc analysis
			*x*	*y*	*z*			
A	R-dorsolateral superior frontal gyrus	1638	24	49.5	28.5	11.629	<0.001∗	SZ<HC (*p* < 0.001∗) BD < HC (P = 0.022∗) SZ vs BD (p = 0.336)
B	L-orbital middle frontal gyrus L-middle frontal gyrus L-orbital superior frontal gyrus	1444	-28.5	60	-2.84	12.008	<0.001∗	SZ<HC (*p* < 0.001∗) BD<HC (*p* = 0.036∗) SZ vs. BD (*p* = 0.191)
C	L-rectus R-rectus	1420	-7.5	36	-21	11.090	<0.001∗	SZ<HC (*p* < 0.001∗) BD<HC (*p* = 0.03∗) SZ vs. BD (*p* = 1.000)
D	R-insula R-temporal pole-superior temporal gyrus R-amygdala	1319	34.5	7.5	-19.5	13.476	<0.001∗	SZ<HC (*p* < 0.001∗) BD<HC (*p* = 0.010∗) SZ vs. BD (*p* = 0.298)
E	L-superior temporal gyrus	943	-61.5	-58.5	15	15.155	<0.001∗	SZ<HC (*p* < 0.001∗) BD vs. HC (*p* = 0.243) SZ<BD (*p* = 0.006∗)
F	L-insula	906	-33	7.5	19.5	12.144	<0.001∗	SZ<HC (*p* < 0.001∗) BD<HC (*p* = 0.017∗) SZ vs. BD (*p* = 0.341)
G	L-dorsolateral superior frontal gyrus L-middle frontal gyrus L-anterior cingulate and paracingulate gyri	856	-19.5	58.5	25.5	15.475	<0.001∗	SZ<HC (*p* < 0.001∗) BD<HC (*p* = 0.006∗) SZ vs. BD (*p* = 0.196)
H	L-insula L-opercular inferior frontal gyrus L-temporal pole-superior temporal gyrus	811	-48	16.5	1.5	14.456	<0.001∗	SZ < HC (P < 0.001∗) BD < HC (P = 0.004∗) SZ vs BD (p = 0.421)
I	R-medial superior frontal gyrus	797	3	60	-1.5	10.622	<0.001∗	SZ<HC (*p* < 0.001∗) BD<HC (*p* = 0.007∗) SZ vs. BD (*p* = 1.000)
J	R-supramarginal gyrus	565	64.5	-16.5	28.5	11.030	<0.001∗	SZ<HC (*p* < 0.001∗) BD vs. HC (*p* = 1.000) SZ<BD (*p* = 0.004∗)

GRF, Gaussian random field corrections; MNI, Montreal Neurological Institute; L, left; R, right; *x*, *y*, *z*, coordinates of peak voxel. ∗Significant at *p* < 0.05. Post hoc analysis is the Bonferroni correction.

## Data Availability

The data that support the findings of this study are available upon reasonable request by contact with the corresponding author, Yanqing Tang.

## Supplementary Materials

Supplementary Materials Table S1: comparison of MCCB scores and cluster values between SZ patients in the first episode and SZ patients not in the first episode. Table S2: comparison of MCCB scores and cluster values between SZ patients with antipsychotic and SZ patients without antipsychotic. Table S3: comparison of MCCB scores and cluster values between SZ patients with mood stabilizer and SZ patients without mood stabilizer. Table S4: comparison of MCCB scores and cluster values between BD patients in the first episode and BD patients not in the first episode. Table S5: comparison of MCCB scores and cluster values between BD patients with antipsychotic and BD patients without antipsychotic. Table S6: comparison of MCCB scores and cluster values between BD patients with mood stabilizer and BD patients without mood stabilizer.

## References

[1] Kahn R. S., Keefe R. S. (2013). Schizophrenia is a cognitive Illness. *JAMA Psychiatry*.

[2] Krabbendam L., Arts B., van Os J., Aleman A. (2005). Cognitive functioning in patients with schizophrenia and bipolar disorder: a quantitative review. *Schizophrenia Research*.

[3] Vöhringer P. A., Barroilhet S., Amerio A. (2013). Cognitive impairment in bipolar disorder and schizophrenia: a systematic review. *Frontiers in Psychiatry*.

[4] Chen C.-K., Lee C.-Y., Lee Y. (2018). Could schizoaffective disorder, schizophrenia and bipolar I disorder be distinguishable using cognitive profiles?. *Psychiatry Research*.

[5] Kim D., Kim J., Koo T., Yun H., Won S. (2015). Shared and distinct neurocognitive endophenotypes of schizophrenia and psychotic bipolar disorder. *Clinical Psychopharmacology and Neuroscience*.

[6] Jensen J. H., Knorr U., Vinberg M., Kessing L. V., Miskowiak K. W. (2016). Discrete neurocognitive subgroups in fully or partially remitted bipolar disorder: associations with functional abilities. *Journal of Affective Disorders*.

[7] Wingo A. P., Harvey P. D., Baldessarini R. J. (2009). Neurocognitive impairment in bipolar disorder patients: functional implications. *Bipolar Disorders*.

[8] Lin P.-Y., Wang P.-W., Chen C.-S., Yen C.-F. (2017). Neurocognitive function in clinically stable individuals with long-term bipolar I disorder: comparisons with schizophrenia patients and controls. *The Kaohsiung Journal of Medical Sciences*.

[9] Depp C. A., Mausbach B. T., Harmell A. L. (2012). Meta-analysis of the association between cognitive abilities and everyday functioning in bipolar disorder. *Bipolar Disorders*.

[10] Nakagawa Y., Chiba K. (2016). Involvement of neuroinflammation during brain development in social cognitive deficits in autism spectrum disorder and schizophrenia. *The Journal of Pharmacology and Experimental Therapeutics*.

[11] Rolstad S., Jakobsson J., Sellgren C. (2015). CSF neuroinflammatory biomarkers in bipolar disorder are associated with cognitive impairment. *European Neuropsychopharmacology*.

[12] Mora E., Portella M. J., Piñol-Ripoll G. (2019). High BDNF serum levels are associated to good cognitive functioning in bipolar disorder. *European Psychiatry*.

[13] Bora E. (2019). Peripheral inflammatory and neurotrophic biomarkers of cognitive impairment in schizophrenia: a meta-analysis. *Psychological Medicine*.

[14] Kubota M., van Haren N. E. M., Haijma S. V. (2015). Association of IQ changes and progressive brain changes in patients with schizophrenia. *JAMA Psychiatry*.

[15] Antonova E., Kumari V., Morris R. (2005). The relationship of structural alterations to cognitive deficits in schizophrenia: a voxel-based morphometry study. *Biological Psychiatry*.

[16] Kubicki M., Shenton M. E., Salisbury D. F. (2002). Voxel-based morphometric analysis of gray matter in first episode schizophrenia. *NeuroImage*.

[17] Koelkebeck K., Dannlowski U., Ohrmann P. (2019). Gray matter volume reductions in patients with schizophrenia: a replication study across two cultural backgrounds. *Psychiatry research. Neuroimaging*.

[18] Lu X., Zhong Y., Ma Z. (2018). Structural imaging biomarkers for bipolar disorder: meta-analyses of whole-brain voxel-based morphometry studies. *Depression and Anxiety*.

[19] Job D. E., Whalley H. C., McConnell S., Glabus M., Johnstone E. C., Lawrie S. M. (2002). Structural gray matter differences between first-episode schizophrenics and normal controls using voxel-based morphometry. *NeuroImage*.

[20] Selvaraj S., Arnone D., Job D. (2012). Grey matter differences in bipolar disorder: a meta-analysis of voxel-based morphometry studies. *Bipolar Disorders*.

[21] Shepherd A. M., Laurens K. R., Matheson S. L., Carr V. J., Green M. J. (2012). Systematic meta-review and quality assessment of the structural brain alterations in schizophrenia. *Neuroscience and Biobehavioral Reviews*.

[22] Wang X., Tian F., Wang S. (2018). Gray matter bases of psychotic features in adult bipolar disorder: a systematic review and voxel-based meta-analysis of neuroimaging studies. *Human Brain Mapping*.

[23] Gutiérrez-Galve L., Bruno S., Wheeler-Kingshott C. A. M., Summers M., Cipolotti L., Ron M. A. (2012). IQ and the fronto-temporal cortex in bipolar disorder. *Journal of the International Neuropsychological Society : JINS*.

[24] Yamada M., Hirao K., Namiki C. (2007). Social cognition and frontal lobe pathology in schizophrenia: a voxel-based morphometric study. *NeuroImage*.

[25] Maila de Castro L. N., Albuquerque M. R., Malloy-Diniz L. (2015). A voxel-based morphometry study of gray matter correlates of facial emotion recognition in bipolar disorder. *Psychiatry Research*.

[26] Davies G., Rae C. L., Garfinkel S. N. (2018). Impairment of perceptual metacognitive accuracy and reduced prefrontal grey matter volume in first-episode psychosis. *Cognitive Neuropsychiatry*.

[27] Delaloye C., de Bilbao F., Moy G. (2009). Neuroanatomical and neuropsychological features of euthymic patients with bipolar disorder. *The American journal of geriatric psychiatry : official journal of the American Association for Geriatric Psychiatry*.

[28] Alonso-Lana S., Goikolea J. M., Bonnin C. M. (2016). Structural and functional brain correlates of cognitive impairment in euthymic patients with bipolar disorder. *PLoS One*.

[29] Wang X., Luo Q., Tian F. (2019). Brain grey-matter volume alteration in adult patients with bipolar disorder under different conditions: a voxel-based meta-analysis. *Journal of psychiatry & neuroscience*.

[30] Hamilton M. (1960). A rating scale for depression. *Journal of Neurology, Neurosurgery, and Psychiatry*.

[31] Young R. C., Biggs J. T., Ziegler V. E., Meyer D. A. (1978). A rating scale for mania: reliability, validity and sensitivity. *The British Journal of Psychiatry*.

[32] Bech P., Larsen J. K., Andersen J. (1988). The BPRS: psychometric developments. *Psychopharmacology Bulletin*.

[33] Bo Q., Mao Z., Li X., Wang Z., Wang C., Ma X. (2017). Use of the MATRICS consensus cognitive battery (MCCB) to evaluate cognitive deficits in bipolar disorder: a systematic review and meta-analysis. *PLoS One*.

[34] Van Rheenen T. E., Rossell S. L. (2014). An empirical evaluation of the MATRICS consensus cognitive battery in bipolar disorder. *Bipolar Disorders*.

[35] Burdick K. E., Goldberg T. E., Cornblatt B. A. (2011). The MATRICS consensus cognitive battery in patients with bipolar I disorder. *Neuropsychopharmacology*.

[36] Sperry S. H., O’Connor L. K., Öngür D., Cohen B. M., Keshavan M. S., Lewandowski K. E. (2015). Measuring cognition in bipolar disorder with psychosis using the MATRICS consensus cognitive battery. *Journal of the International Neuropsychological Society*.

[37] Ashburner J., Friston K. J. (2000). Voxel-based morphometry--the methods. *NeuroImage*.

[38] Ashburner J. (2007). A fast diffeomorphic image registration algorithm. *NeuroImage*.

[39] Jiménez-López E., Aparicio A. I., Sánchez-Morla E. M., Rodriguez-Jimenez R., Vieta E., Santos J. L. (2017). Neurocognition in patients with psychotic and non-psychotic bipolar I disorder. A comparative study with individuals with schizophrenia. *Journal of affective disorders*.

[40] Gold J. M., Barch D. M., Feuerstahler L. M. (2019). Working memory impairment across psychotic disorders. *Schizophrenia Bulletin*.

[41] Ishisaka N., Shimano S., Miura T. (2017). Neurocognitive profile of euthymic Japanese patients with bipolar disorder. *Psychiatry and Clinical Neurosciences*.

[42] Czepielewski L. S., Massuda R., Goi P. (2015). Verbal episodic memory along the course of schizophrenia and bipolar disorder: a new perspective. *European neuropsychopharmacology : the journal of the European College of Neuropsychopharmacology*.

[43] Sheffield J., Karcher N., Barch D. (2018). Cognitive deficits in psychotic disorders: a lifespan perspective. *Neuropsychology Review*.

[44] Van Rheenen T. E., Cropley V., Zalesky A. (2018). Widespread volumetric reductions in schizophrenia and schizoaffective patients displaying compromised cognitive abilities. *Schizophrenia Bulletin*.

[45] Woodward N., Heckers S. (2015). Brain structure in neuropsychologically defined subgroups of schizophrenia and psychotic bipolar disorder. *Schizophrenia Bulletin*.

[46] Maggioni E., Bellani M., Altamura A. C., Brambilla P. (2016). Neuroanatomical voxel-based profile of schizophrenia and bipolar disorder. *Epidemiology and Psychiatric Sciences*.

[47] Brown G. G., Lee J.-S., Strigo I. A., Caligiuri M. P., Meloy M. J., Lohr J. (2011). Voxel-based morphometry of patients with schizophrenia or bipolar I disorder: a matched control study. *Psychiatry Research*.

[48] Nenadic I., Maitra R., Langbein K. (2015). Brain structure in schizophrenia vs. psychotic bipolar I disorder: a VBM study. *Schizophrenia Research*.

[49] James A., Hough M., James S. (2011). Structural brain and neuropsychometric changes associated with pediatric bipolar disorder with psychosis. *Bipolar Disorders*.

[50] Liu S., Wang H., Song M. (2019). Linked 4-way multimodal brain differences in schizophrenia in a large Chinese Han population. *Schizophrenia Bulletin*.

[51] Vargas T., Dean D. J., Osborne K. J. (2018). Hippocampal subregions across the psychosis spectrum. *Schizophrenia Bulletin*.

[52] Hidese S., Ota M., Matsuo J. (2017). Association between the scores of the Japanese version of the brief assessment of cognition in schizophrenia and whole-brain structure in patients with chronic schizophrenia: a voxel-based morphometry and diffusion tensor imaging study. *Psychiatry and Clinical Neurosciences*.

[53] Keshavan M. S., Morris D. W., Sweeney J. A. (2011). A dimensional approach to the psychosis spectrum between bipolar disorder and schizophrenia: the schizo-bipolar scale. *Schizophrenia Research*.

[54] Jimenez A. M., Riedel P., Lee J., Reavis E. A., Green M. F. (2019). Linking resting-state networks and social cognition in schizophrenia and bipolar disorder. *Human Brain Mapping*.

