# Supplementary material for: Distinct Associations of Cognitive Impairments and Reduced Gray Matter Volumes in Remitted Patients with Schizophrenia and Bipolar Disorder
Source: Neural Plast. 2020 Dec 10;2020:8859388. doi: 10.1155/2020/8859388 (PMC7748913; doi:10.1155/2020/8859388)
Supplement: Supplementary Materials — Table S1: comparison of MCCB scores and cluster values between SZ patients in the first episode and SZ patients not in the first episode. Table S2: comparison of MCCB scores and cluster values between SZ patients with antipsychotic and SZ patients without antipsychotic. Table S3: comparison of MCCB scores and cluster values between SZ patients with mood stabilizer and SZ patients without mood stabilizer. Table S4: comparison of MCCB scores and cluster values between BD patients in the first episode and BD patients not in the first episode. Table S5: comparison of MCCB scores and cluster values between BD patients with antipsychotic and BD patients without antipsychotic. Table S6: comparison of MCCB scores and cluster values between BD patients with mood stabilizer and BD patients without mood stabilizer. [file 8859388.f1.docx]

Table S1: Comparison of MCCB scores and Cluster values between SZ patients in the first episode and SZ patients not in the first episode.

|  | Group; mean ± SD | |  |  |
| --- | --- | --- | --- | --- |
| Characteristic | SZ_first episode  （n=27） | SZ_no first episode  （n=19） | t | p value |
| MCCB scores | | | | |
| composite score | 43.59±9.02 | 37.89±11.35 | 1.895 | 0.065 |
| TMT-A | 38.26±9.94 | 51.16±17.62 | -2.884 | 0.008* |
| Symbol Coding | 51.07±14.88 | 46.53±12.21 | 1.097 | 0.279 |
| HVLT-R | 25.33±5.24 | 22.63±5.85 | 1.641 | 0.108 |
| Spatial Span | 15.85+3.88 | 13.58+4.93 | 1.750 | 0.087 |
| Letter Number Span | 21.96+3.08 | 18.89±4.04 | 2.923 | 0.005* |
| Mazes | 11.26±5.48 | 10.68±5.54 | 0.349 | 0.729 |
| BVMT-R | 24.48±7.39 | 20.89±7.47 | 1.614 | 0.114 |
| Category Fluency | 20.59±4.84 | 19.63±7.06 | 0.548 | 0.586 |
| MECEIT | 8.42±2.06 | 8.08±2.00 | 0.560 | 0.578 |
| CPT | 0.32±0.62 | -0.03±0.94 | 1.521 | 0.135 |
| Cluster values | | | | |
| Cluster_A | 0.50±0.08 | 0.51±0.05 | -0.291 | 0.772 |
| Cluster_B | 0.51±0.07 | 0.53±0.05 | -1.061 | 0.294 |
| Cluster_C | 0.55±0.06 | 0.57±0.05 | -0.671 | 0.506 |
| Cluster_D | 0.58±0.04 | 0.58±0.05 | -0.124 | 0.902 |
| Cluster_E | 0.43±0.05 | 0.44±0.04 | -1.296 | 0.202 |
| Cluster_F | 0.58±0.05 | 0.58±0.05 | 0.042 | 0.967 |
| Cluster_G | 0,52±0.07 | 0,52±0,05 | -0.339 | 0.736 |
| Cluster_H | 0.58±0.05 | 0.57±0.06 | 0.744 | 0.461 |
| Cluster_I | 0.46±0.05 | 0.47±0.05 | -0.574 | 0.569 |
| Cluster_J | 0.59±0.09 | 0.59±0.07 | -0.006 | 0.995 |
| t = Independent-samples t-test; TMT-A = Trail Making Test A; HVLT-R = Hopkins Verbal Learning Test - Revised; BVMT-R = Brief Visuospatial Memory Test - Revised; MSCEIT = Mayer-Salovey-Caruso Emotional Intelligence Test; CPT-IP = Continuous Performance Test - Identical Pairs.  *Significant at p < 0.05 | | | | |

Table S2: Comparison of MCCB scores and Cluster values between SZ patients with antipsychotic and SZ patients without antipsychotic.

|  | Group; mean ± SD | |  |  |
| --- | --- | --- | --- | --- |
| Characteristic | SZ_antipsychotic  （n=38） | SZ_no antipsychotic  （n=8） | t | p value |
| MCCB scores | | | | |
| composite score | 41.16±10.21 | 41.63±11.60 | -0.115 | 0.909 |
| TMT-A | 42.50±13.89 | 48.75±19.36 | -1.079 | 0.287 |
| Symbol Coding | 50.29±14.03 | 44.00±12.66 | 1.170 | 0.248 |
| HVLT-R | 24.24±5.40 | 24.13±6.90 | 0.051 | 0.960 |
| Spatial Span | 15.58±3.74 | 11.75±6.23 | 2.325 | 0.025* |
| Letter Number Span | 20.79±3.81 | 20.25±3.88 | 0.363 | 0.719 |
| Mazes | 11.34±5.80 | 9.50±3.21 | 1.251 | 0.227 |
| BVMT-R | 23.74±7.69 | 19.50±6.14 | 1.460 | 0.151 |
| Category Fluency | 20.21±5.89 | 20.13±5.79 | 0.037 | 0.970 |
| MECEIT | 8.07±2.02 | 9.25±1.78 | -1.533 | 0.132 |
| CPT | 0.16±0.80 | 0.24±0.72 | -0.279 | 0.782 |
| Cluster values | | | | |
| Cluster_A | 0.51±0.07 | 0.51±0.05 | -0.177 | 0.860 |
| Cluster_B | 0.52±0.06 | 0.53±0.07 | -0.719 | 0.476 |
| Cluster_C | 0.56±0.06 | 0.57±0.06 | -0.629 | 0.533 |
| Cluster_D | 0.57±0.04 | 0.59±0.05 | -0.836 | 0.408 |
| Cluster_E | 0.43±0.05 | 0.46±0.03 | -1.692 | 0.098 |
| Cluster_F | 0.57±0.05 | 0.59±0.04 | -0.771 | 0.445 |
| Cluster_G | 0.52±0.06 | 0.53±0.06 | -0.638 | 0.527 |
| Cluster_H | 0.58±0.07 | 0.58±0.09 | 0.021 | 0.983 |
| Cluster_I | 0.47±0.06 | 0.48±0.06 | -0.502 | 0.618 |
| Cluster_J | 0.59±0.08 | 0.60±0.09 | -0.513 | 0.611 |
| t = Independent-samples t-test; TMT-A = Trail Making Test A; HVLT-R = Hopkins Verbal Learning Test - Revised; BVMT-R = Brief Visuospatial Memory Test - Revised; MSCEIT = Mayer-Salovey-Caruso Emotional Intelligence Test; CPT-IP = Continuous Performance Test - Identical Pairs.  *Significant at p < 0.05 | | | | |

Table S3: Comparison of MCCB scores and Cluster values between SZ patients with mood stabilizer and SZ patients without mood stabilizer.

|  | Group; mean ± SD | |  |  |
| --- | --- | --- | --- | --- |
| Characteristic | SZ_ mood stabilizer  （n=7） | SZ_no mood stabilizer  （n=39） | t | p value |
| MCCB scores | | | | |
| composite score | 46.71±11.09 | 40.26±10.02 | 1.547 | 0.129 |
| TMT-A | 45.57±20.63 | 43.23±13.98 | 0.379 | 0.707 |
| Symbol Coding | 53.71±15.64 | 48.38±13.61 | 0.934 | 0.355 |
| HVLT-R | 26.14±4.49 | 23.87±5.76 | 0.987 | 0.329 |
| Spatial Span | 14.43±1.90 | 15.00±4.76 | -0.311 | 0.758 |
| Letter Number Span | 22.14±2.41 | 20.44±3.95 | 1.100 | 0.277 |
| Mazes | 13.14±5.55 | 10.64±5.42 | 1.121 | 0.268 |
| BVMT-R | 24.86±6.72 | 22.67±7.73 | 0.702 | 0.486 |
| Category Fluency | 20.14±7.34 | 20.21±5.61 | -0.026 | 0.980 |
| MECEIT | 8.43±1.97 | 8.25±2.05 | 0.220 | 0.827 |
| CPT | 0.52±0.80 | 0.11±0.77 | 1.316 | 0.195 |
| Cluster values | | | | |
| Cluster_A | 0.45±0.04 | 0.52±0.06 | -2.908 | 0.006* |
| Cluster_B | 0.46±0.05 | 0.53±0.06 | -3.152 | 0.003* |
| Cluster_C | 0.51±0.04 | 0.57±0.06 | -2.666 | 0.011* |
| Cluster_D | 0.54±0.03 | 0.58±0.04 | -2.639 | 0.011* |
| Cluster_E | 0.41±0.02 | 0.44±0.05 | -1.386 | 0.173 |
| Cluster_F | 0.54±0.02 | 0.58±0.05 | -2.387 | 0.021* |
| Cluster_G | 0.48±0.05 | 0.53±0.06 | -1.736 | 0.090 |
| Cluster_H | 0.53±0.08 | 0.58±0.07 | -1.828 | 0.074 |
| Cluster_I | 0.41±0.04 | 0.48±0.05 | -3.655 | 0.001* |
| Cluster_J | 0.54±0.04 | 0.60±0.08 | -1.977 | 0.054 |
| t = Independent-samples t-test; TMT-A = Trail Making Test A; HVLT-R = Hopkins Verbal Learning Test - Revised; BVMT-R = Brief Visuospatial Memory Test - Revised; MSCEIT = Mayer-Salovey-Caruso Emotional Intelligence Test; CPT-IP = Continuous Performance Test - Identical Pairs.  *Significant at p < 0.05 | | | | |

Table S4: Comparison of MCCB scores and Cluster values between BD patients in the first episode and BD patients not in the first episode.

|  | Group; mean ± SD | |  |  |
| --- | --- | --- | --- | --- |
| Characteristic | BD_first episode  （n=10） | BD_no first episode  （n=25） | t | p value |
| MCCB scores | | | | |
| composite score | 50.60±9.42 | 47.12±9.63 | 0.971 | 0.338 |
| TMT-A | 36.50±16.79 | 48.88±33.86 | -1.096 | 0.281 |
| Symbol Coding | 53.70±13.03 | 52.56±16.85 | 0.192 | 0.849 |
| HVLT-R | 29.80±2.70 | 28.20±4.75 | 0.997 | 0.326 |
| Spatial Span | 17.60±5.25 | 15.40±5.55 | 1.074 | 0.290 |
| Letter Number Span | 25.30±2.63 | 20.64±4.89 | 3.632 | 0.001* |
| Mazes | 14.10±8.08 | 14.52±7.49 | -0.147 | 0.884 |
| BVMT-R | 27.20±8.47 | 25.88±7.42 | 0.457 | 0.651 |
| Category Fluency | 23.10±13.78 | 21.32±5.70 | 0.395 | 0.701 |
| MECEIT | 8.28±1.61 | 9.11±1.56 | -1.407 | 0.169 |
| CPT | 0.76±0.34 | 0.70±0.57 | 0.390 | 0.699 |
| Cluster values | | | | |
| Cluster_A | 0.57±0.08 | 0.52±0.07 | 1.778 | 0.085 |
| Cluster_B | 0.56±0.05 | 0.54±0.06 | 1.117 | 0.272 |
| Cluster_C | 0.59±0.03 | 0.56±0.05 | 2.028 | 0.051 |
| Cluster_D | 0.63±0.03 | 0.58±0.05 | 2.745 | 0.010* |
| Cluster_E | 0.48±0.02 | 0.46±0.05 | 1.492 | 0.145 |
| Cluster_F | 0.62±0.05 | 0.58±0.06 | 1.974 | 0.057 |
| Cluster_G | 0.58±0.05 | 0.53±0.06 | 2.303 | 0.028* |
| Cluster_H | 0.62±0.07 | 0.60±0.08 | 0.722 | 0.476 |
| Cluster_I | 0.51±0.05 | 0.47±0.06 | 1.841 | 0.075 |
| Cluster_J | 0.68±0.05 | 0.64±0.09 | 1.715 | 0.096 |
| t = Independent-samples t-test; TMT-A = Trail Making Test A; HVLT-R = Hopkins Verbal Learning Test - Revised; BVMT-R = Brief Visuospatial Memory Test - Revised; MSCEIT = Mayer-Salovey-Caruso Emotional Intelligence Test; CPT-IP = Continuous Performance Test - Identical Pairs.  *Significant at p < 0.05 | | | | |

Table S5: Comparison of MCCB scores and Cluster values between BD patients with antipsychotic and BD patients without antipsychotic.

|  | Group; mean ± SD | |  |  |
| --- | --- | --- | --- | --- |
| Characteristic | BD_antipsychotic  （n=17） | BD_no antipsychotic  （n=18） | t | p value |
| MCCB scores | | | | |
| composite score | 43.71±8.60 | 52.28±8.70 | -2.930 | 0.006* |
| TMT-A | 53.82±38.87 | 37.33±16.45 | 1.618 | 0.120 |
| Symbol Coding | 52.24±14.87 | 53.50±16.80 | -0.235 | 0.815 |
| HVLT-R | 26.88±4.15 | 30.33±3.80 | -2.566 | 0.015* |
| Spatial Span | 13.82±6.44 | 18.11±3.41 | -2.439 | 0.022* |
| Letter Number Span | 19.76±5.30 | 24.06±3.26 | -2.865 | 0.008* |
| Mazes | 11.94±8.12 | 16.72±6.32 | -1.950 | 0.060 |
| BVMT-R | 24.35±9.29 | 28.06±5.32 | -1.436 | 0.163 |
| Category Fluency | 20.24±6.77 | 23.33±9.98 | -1.068 | 0.293 |
| MECEIT | 8.88±1.43 | 8.87±1.78 | 0.011 | 0.992 |
| CPT | 0.64±0.53 | 0.78±0.49 | -0.814 | 0.422 |
| Cluster values | | | | |
| Cluster_A | 0.52±0.08 | 0.54±0.08 | -0.879 | 0.386 |
| Cluster_B | 0.54±0.06 | 0.56±0.05 | -1.062 | 0.296 |
| Cluster_C | 0.56±0.06 | 0.58±0.04 | -0.921 | 0.364 |
| Cluster_D | 0.58±0.06 | 0.61±0.04 | -2.224 | 0.035* |
| Cluster_E | 0.46±0.05 | 0.47±0.04 | -1.102 | 0.278 |
| Cluster_F | 0.58±0.06 | 0.61±0.05 | -1.336 | 0.191 |
| Cluster_G | 0.53±0.07 | 0.56±0.05 | -1.199 | 0.239 |
| Cluster_H | 0.57±0.07 | 0.63±0.06 | -2.888 | 0.007* |
| Cluster_I | 0.46±0.06 | 0.50±0.05 | -2.360 | 0.024* |
| Cluster_J | 0.64±0.09 | 0.65±0.07 | -0.425 | 0.673 |
| t = Independent-samples t-test; TMT-A = Trail Making Test A; HVLT-R = Hopkins Verbal Learning Test - Revised; BVMT-R = Brief Visuospatial Memory Test - Revised; MSCEIT = Mayer-Salovey-Caruso Emotional Intelligence Test; CPT-IP = Continuous Performance Test - Identical Pairs.  *Significant at p < 0.05 | | | | |

Table S6: Comparison of MCCB scores and Cluster values between BD patients with mood stabilizer and BD patients without mood stabilizer.

|  | Group; mean ± SD | |  |  |
| --- | --- | --- | --- | --- |
| Characteristic | BD_ mood stabilizer  （n=17） | BD_no mood stabilizer  （n=18） | t | p value |
| MCCB scores | | | | |
| composite score | 45.35±8.70 | 50.72±9.85 | -1.706 | 0.097 |
| TMT-A | 54.71±37.99 | 36.50±17.42 | 1.840 | 0.075 |
| Symbol Coding | 52.53±13.54 | 53.22±17.84 | -0.129 | 0.898 |
| HVLT-R | 27.47±4.30 | 29.78±4.08 | -1.628 | 0.113 |
| Spatial Span | 14.47±5.91 | 17.50±4.76 | -1.676 | 0.103 |
| Letter Number Span | 20.41±4.91 | 23.44±4.37 | -1.932 | 0.062 |
| Mazes | 12.59±7.67 | 16.11±7.21 | -1.400 | 0.171 |
| BVMT-R | 25.65±8.94 | 26.83±6.36 | -0.454 | 0.653 |
| Category Fluency | 21.24±6.35 | 22.39±10.44 | -0.392 | 0.698 |
| MECEIT | 9.34±1.28 | 8.43±1.77 | 1.737 | 0.092 |
| CPT | 0.57±0.52 | 0.85±0.47 | -1.701 | 0.098 |
| Cluster values | | | | |
| Cluster_A | 0.52±0.09 | 0.54±0.07 | -0.818 | 0.419 |
| Cluster_B | 0.54±0.06 | 0.55±0.05 | -0.277 | 0.784 |
| Cluster_C | 0.57±0.06 | 0.57±0.04 | -0.182 | 0.857 |
| Cluster_D | 0.58±0.06 | 0.60±0.03 | -1.216 | 0.236 |
| Cluster_E | 0.47±0.05 | 0.46±0.04 | 0.479 | 0.635 |
| Cluster_F | 0.59±0.06 | 0.60±0.05 | -0.640 | 0.526 |
| Cluster_G | 0.54±0.07 | 0.54±0.05 | 0.018 | 0.986 |
| Cluster_H | 0.58±0.08 | 0.62±0.07 | -1.373 | 0.179 |
| Cluster_I | 0.47±0.06 | 0.49±0.06 | -0.858 | 0.397 |
| Cluster_J | 0.65±0.09 | 0.65±0.07 | -0.011 | 0.991 |
| t = Independent-samples t-test; TMT-A = Trail Making Test A; HVLT-R = Hopkins Verbal Learning Test - Revised; BVMT-R = Brief Visuospatial Memory Test - Revised; MSCEIT = Mayer-Salovey-Caruso Emotional Intelligence Test; CPT-IP = Continuous Performance Test - Identical Pairs.  *Significant at p < 0.05 | | | | |
